# Diseases of the Spinal Cord

**Published:** 1886-08

**Authors:** 


DISEASES OF THE SPINAL CORD. By Byrom Bromwell, M. D., F. R. C. P. (Edin.), Lecturer on the Principles and Practice of Medicine, and on Medical Diagnosis in the extra Academical School of Medicine, Edinburgh; Pathologist to the Edinburgh Royal Infirmary; Additional Examiner in Clinical Medicine in the University of Edinburgh; Late Physician and Pathologist to the New Castle-on-Tyne Infirmary; formerly Medical Officer to the Tynemouth Union Workhouse Hospital, the Prudhoe Memorial Convalescent Home, the Tyne Floating Hospital, etc. Fifty-three colored plates and one hundred and two fine wood engravings. Second edition. New York: Wm. Wood & Co. 1886.

This is the January number of Wood's Library of Standard Medical Authors and is a valuable contribution to the literature of the subject. The many obscure affections of the spinal cord, which have been left by the general practitioner for the specialist, may be well understood and properly treated after becoming familiar with this work. The fact that it has already been translated into the German, French and Russian languages attests its merits. The work is profusely illustrated by wood engravings and colored plates which adds very much to its value. The entire work of the publishers is excellent, and they are entitled to the thanks of the profession for their efforts to give them standard medical literature at a mere nominal cost.

